# Region-specific glucocorticoid receptor promoter methylation has both positive and negative prognostic value in patients with estrogen receptor-positive breast cancer

**DOI:** 10.1186/s13148-019-0750-x

**Published:** 2019-11-01

**Authors:** Hilary Snider, Brithica Villavarajan, Yingwei Peng, Lois E. Shepherd, Andrew C. Robinson, Christopher R. Mueller

**Affiliations:** 1 Division of Cancer Biology and Genetics, Queen’s Cancer Research Institute, Queen’s University, Kingston, Ontario Canada; 2 Department of Pathology and Molecular Medicine, Queen’s University, Kingston, Ontario Canada; 3 Division of Cancer Care and Epidemiology, Queen’s Cancer Research Institute, Queen’s University, Kingston, Ontario Canada; 4 Department of Mathematics and Statistics, Queen’s University, Kingston, Ontario Canada; 5 Department of Public Health Sciences, Queen’s University, Kingston, Ontario Canada; 6 Canadian Cancer Trials Group, Queen’s University, Kingston, Canada; 7 Department of Oncology, Division of Medical Oncology, Queen’s University, Kingston, Canada; 8 Department of Biomedical and Molecular Sciences, Queen’s University, Kingston, Ontario Canada

**Keywords:** Breast cancer, Glucocorticoid receptor, DNA methylation, Prognosis, Bisulfite sequencing

## Abstract

**Background:**

The glucocorticoid receptor (*NR3C1*, GR) is frequently downregulated in breast tumors, and evidence suggests it acts as a tumor suppressor in estrogen receptor-positive (ER+) breast cancer. We previously found that methylation of the GR promoter CpG island represses gene expression and occurs in ER+ breast tumors. In this study, the prognostic and predictive value of GR methylation was examined in ER+ patients from the CCTG MA.12 clinical trial of tamoxifen versus placebo in women with early breast cancer.

**Methods:**

We developed a targeted multiplex bisulfite next-generation sequencing assay to detect methylation at multiple GR promoter regions in DNA from formalin-fixed paraffin-embedded (FFPE) samples. Following validation in a small cohort of breast tumors, ER+ FFPE tumor samples from MA.12 (*n* = 208) were tested. Survival analyses evaluated the impact of GR promoter methylation on patient overall survival (OS) and disease-free survival (DFS).

**Results:**

An analysis of TCGA data found that GR methylation is prevalent in ER+ tumors and is associated with decreased gene expression and analysis of public microarray data (KM Plotter) linked decreased GR expression to a poor outcome. In MA.12, two GR promoter regions (U and C) each had prognostic value, but with opposite effects on the outcome. U methylation was associated with poor OS (HR = 1.79, *P* = 0.041) whereas C methylation was associated with better OS (HR = 0.40, *P* = 0.040) and DFS (HR = 0.49, *P* = 0.037). The classification of patients based on the methylation status of the two regions was prognostic for OS (*P* = 0.006) and DFS (*P* = 0.041) and revealed a group of patients (U methylated, C unmethylated) with very poor outcomes. Placebo-treated patients in this high-risk group had worse OS (HR = 2.86, *P* = 0.002) and DFS (HR = 2.09, *P* = 0.014) compared to the rest of the cohort.

**Conclusion:**

Region-specific GR promoter methylation was an independent prognostic marker for patient survival and identified a subset of patients with poor prognosis, particularly without tamoxifen treatment. These findings provide a foundation for future studies into GR methylation as a promising prognostic biomarker in ER+ breast cancer.

## Background

Breast cancer is a heterogeneous disease, so determining an accurate and specific prognosis is essential to ensure that each patient receives the most effective course of treatment. A prognosis is typically based on a combination of clinical characteristics (patient age, tumor size, tumor grade, and lymph node involvement) and histopathological features such as hormone receptor status [1, 2]. The majority of breast cancers express the estrogen receptor (ER) and are therefore responsive to endocrine therapies that impair estrogen signaling. Tamoxifen, a selective estrogen receptor modulator, has been the standard of care for ER+ patients for over 30 years. Its use has reduced recurrence rates by nearly one-half and has decreased disease mortality by about one-third [3–5]. Nevertheless, around 1 in 4 women treated with tamoxifen relapse within 10 years of treatment [3, 6]; thus, resistance to endocrine therapy is a major clinical concern and it is evident that ER status alone is insufficient to predict treatment response. Several multigene expression profiling assays have been developed as prognostic tools for the ER+ patient population including OncotypeDX, Prosigna (PAM50-based), and Mammaprint, all of which are in clinical use [2, 7, 8]. Although these tests further stratify ER+ patients by recurrence risk, their primary utility is to identify individuals who can safely be spared chemotherapy and they do not usually influence endocrine therapy treatment decisions [2, 7, 8]. Due to the limitations of current prognostic and predictive tools, there is an urgent need for the development of robust markers that can predict patient response to therapy prior to them undergoing treatment, allowing for timely interventions to minimize their risk of relapse and maximize survival.

The failure of endocrine therapies like tamoxifen can often be attributed to factors that modify or bypass the ER signaling pathway. De novo or pre-existing ER mutants account for a significant fraction of therapy failures, but the majority are due to other causes [9]. Recently, several studies have demonstrated that the expression and activation of the glucocorticoid receptor (GR) have a direct impact on the regulation of many ER target genes (reviewed in [10–12]). This is mediated by the crosstalk that occurs between GR and ER in their role as transcriptional regulators. The coactivation of both receptors leads to the recruitment of GR to ER DNA binding sites, which can have both cooperative and antagonistic gene-specific outcomes [13–16]. In ER+ breast cancer cells that express GR, the activation of both receptors, as opposed to ER alone, results in the increased expression of pro-differentiation genes which are associated with improved relapse-free survival in ER+ patients [13]. GR can also impede estrogen-stimulated cell growth by directly blocking the recruitment of transcriptional coactivators to ER-bound enhancers, repressing the transcription of estrogen-activated genes [17]. Overall, it appears that by modulating ER-directed gene transcription, GR promotes a more indolent tumor phenotype in ER+ breast cancers. The loss of this effect could permit cells to continue to survive and proliferate despite being subjected to ER-targeted therapies, leading to poor treatment response. Indeed, studies have found that low GR expression in ER+ breast cancer is associated with worse patient outcomes [13, 18].

Multiple studies have established that GR protein is frequently decreased in breast tumors in comparison to normal tissue [19–23], and lower GR expression is associated with higher tumor grade [20, 21]. We have previously identified that methylation of the GR gene (*NR3C1*) promoter is a common event that contributes to the downregulation of GR in breast cancer [24]. We found that while the GR promoter is not methylated in normal mammary tissue, it is methylated in approximately 15% of breast tumors. These GR-methylated tumors exhibited particularly low GR expression and were predominantly ER+. Given that methylation influences GR expression and that low GR-expressing tumors have been associated with poor relapse-free survival in ER+ tamoxifen-treated patients [13, 18], we propose that GR methylation may act as a biomarker for increased relapse risk and/or poor survival. In this study, we developed a multiplex bisulfite sequencing assay for detecting GR promoter methylation and used it to investigate the prognostic and predictive significance of GR methylation in a cohort of ER+ patients from a placebo-controlled trial of adjuvant tamoxifen in premenopausal women with early breast cancer (CCTG MA.12). This study is an excellent model for the analysis of methylation in archival clinical trial material.

## Methods

### MA.12 patient cohort

The Canadian Cancer Trials Group (CCTG) MA.12 study was a randomized placebo-controlled phase III trial of adjuvant tamoxifen for 5 years in premenopausal women with high-risk early breast cancer. The MA.12 trial was approved by local research ethics boards (Queen’s University DBMS-049-15) and participants provided a written informed consent. Between 1993 and 2000, 672 women were enrolled in MA.12. The median age was 46 years (range 29–58). After surgery, patients received standard adjuvant chemotherapy (cyclophosphamide/methotrexate/5-fluorouracil (CMF), cyclophosphamide/epirubicin/5-fluorouracil (CEF), or doxorubicin (adriamycin)/cyclophosphamide (AC)) followed by randomization to tamoxifen or placebo for 5 years. Levels of at least one hormone receptor (ER and/or PR) were determined by biochemical or immunohistochemical methods, but patients were eligible regardless of receptor status. The clinical endpoints were overall survival (OS) and disease-free survival (DFS). OS was defined as the time from randomization to date of death or censored on the last date the patient was known to be alive, and DFS was defined as the time from randomization to the earliest date of recurrence or death or censored on the last date the patient was known to be alive. The median follow-up of the study was 9.7 years. The details on the conduct of this study and its results have been published [25].

Formalin-fixed, paraffin-embedded (FFPE) blocks were requested for all 454 ER+ patients from the study and a total of 252 tumor samples were available. Samples that did not pass the DNA extraction criteria (DNA concentration > 5 ng/μL) were excluded (*n* = 43), as was one sample with missing outcome data. This resulted in a final study cohort of 208 patients, the characteristics of which are summarized in Table 1.

**Table 1:** Patient cohort characteristics

Characteristic	All ER+ MA.12 patients		GR methylation study cohort	
	(*N* = 454)		(*N* = 208)	
	*N*	%	*N*	%
Age				
≤ 45 years	268	59.0	125	60.1
> 45 years	186	41.0	83	39.9
Stage (pathological)				
I	40	8.8	12	5.8
II	387	85.2	180	86.5
III	27	5.9	16	7.7
Tumor stage (T-stage)				
T1	212	46.7	90	43.3
T2	213	46.9	102	49.0
T3/T4	29	6.4	16	7.7
Nodal status				
Node negative	87	19.2	28	13.5
1–3 nodes	276	60.8	130	62.5
4–9 nodes	78	17.2	42	20.2
10+ nodes	13	2.9	8	3.8
Adjuvant chemotherapy				
CEF	104	22.9	52	25.0
CMF	197	43.4	89	42.8
AC	153	33.7	67	32.2
Treatment				
Placebo	231	50.9	112	53.8
Tamoxifen	223	49.1	96	46.2
Outcome				
Deaths	107	23.6	56	26.9
Recurrences	154	33.9	86	41.3

### DNA extraction and bisulfite conversion

Archival FFPE blocks from the MA.12 clinical trial had three 1.0 mm tissue cores extracted for DNA isolation. Cores were deparaffinized with xylene and rinsed with 100% ethanol, and DNA was extracted with the AllPrep DNA/RNA FFPE Kit (Qiagen) as per manufacturer’s instructions. Quantitation of DNA yield was performed with a Qiaxpert spectrophotometer (Qiagen), and samples with a minimum DNA concentration of 5 ng/μL (140 ng total yield) were subjected to bisulfite conversion.

DNA was extracted from the MCF-7 and T47-D human breast cancer cell lines using the GenElute Mammalian Genomic DNA Mini-Prep Kit (Sigma-Aldrich) as described by the manufacturer. The isolation of DNA from MCF-7 and T47-D FFPE cell pellets was performed using the same methods as the MA.12 samples, outlined above. DNA from the Ontario Tumor Bank fresh frozen breast tumor samples used in this study was extracted previously, as described in [24]. Briefly, tumor samples were completely homogenized, and DNA extraction was carried out using the AllPrep DNA/RNA Mini Kit (Qiagen).

All DNA samples in this study were bisulfite converted using the EpiTect Fast DNA Bisulfite Kit (Qiagen) following the manufacturer’s protocol.

### Quantitative methylation-specific PCR

Quantitative methylation-specific PCR (qMSP) was conducted using primer sets (Additional file 1: Table S1, hg19 genomic coordinates in Additional file 2: Table S2) designed using MethPrimer [26] to specifically amplify either the methylated or unmethylated bisulfite-converted target sequence. For each reaction, 10 ng bisulfite-converted DNA was amplified using 1X QuantiTect SYBR Green PCR Master Mix (Qiagen) and 50 ng forward and reverse primers in a 20 μL volume. The assay was performed in an Applied Biosystems ViiA 7 Real-Time PCR System (Life Technologies) with the following conditions: 95 °C for 15 min, followed by 40 cycles of denaturing at 95 °C for 30 s, annealing at 58 °C for 30 s, and elongation at 72 °C for 30 s. Cycle threshold (Ct) values were obtained for both the methylated-specific (M) and unmethylated-specific (U) primer sets and percent methylation was calculated for each sample using the following formula: % methylation = 100/(1 + 2^ΔCt(M-U)^) %. PCR products were visualized by running 4 μL sample on 2.5% agarose gels.

### Library construction and bisulfite sequencing

Bisulfite sequencing libraries were created using a two-step PCR process. Primary PCR (singleplex or multiplex) was performed with GR bisulfite sequencing primers (Table 2) to amplify target GR promoter regions (Additional file 2: Table S2), followed by a secondary PCR for the addition of barcoded sequencing adapters.

**Table 2:** Primers for GR bisulfite sequencing

Primer	Primer sequence (5′ to 3′)	*NR3C1* target region [27]	# Interrogated CpGs	Amplicon size (bp)
A	Fwd: ACACTGACGACATGGTTCTACAGGAGGGTGGGTTTTGTTTTGTAAT	− 3664 to − 3471	17	193
	Rev: TACGGTAGCAGAGACTTGGTCTACCTAACACRCCCTCTAAAAAAAC			
C	Fwd: ACACTGACGACATGGTTCTACATTTTTTATTTTGYGAGTTYGTGTTTGT	− 2754 to − 2633	9	121
	Rev: TACGGTAGCAGAGACTTGGTCTCCCRATCCCAACTACTTCRACC			
D	Fwd: ACACTGACGACATGGTTCTACAGGGTGGAAGGAGAYGTYGTAGT	− 2682 to − 2532	16	150
	Rev: TACGGTAGCAGAGACTTGGTCTAAACCCCTATTTAAAAAAATCTCCCA			
F	Fwd: ACACTGACGACATGGTTCTACAATTTTTATTAGTTTYGGGGAGTGGG	− 2275 to − 2125	11	150
	Rev: TACGGTAGCAGAGACTTGGTCTCCRAAATCAAATTCCTCCCCCTC			
L	Fwd: ACACTGACGACATGGTTCTACATTTTYGAAGTGATATATTTTAYGTAATT	− 3410 to − 3251	17	159
	Rev: TACGGTAGCAGAGACTTGGTCTRAAAACTCRCTCTACCCCTTAAC			
P	Fwd: ACACTGACGACATGGTTCTACAGAAYGTGATAGGGTGAGTAAYGTA	− 4062 to − 3910	7	152
	Rev: TACGGTAGCAGAGACTTGGTCTAATTACTAACRAAATATAACCCCCCT			
T	Fwd: ACACTGACGACATGGTTCTACATGAGAATTAAGGAAGGAYGGTTTAG	− 4684 to − 4535	12	149
	Rev: TACGGTAGCAGAGACTTGGTCTAACATCTTAAAAACRATTAAAAAAACRC			
U	Fwd: ACACTGACGACATGGTTCTACAGTTAAGTTGTTTATTTYGGTTGYGG	− 2440 to − 2304	13	136
	Rev: TACGGTAGCAGAGACTTGGTCTTATCTCCRATCCCAACRACACCT			
W	Fwd: ACACTGACGACATGGTTCTACAGTAGGGGGAGTYGTYGTTAGTTT	− 4531 to − 4381	10	150
	Rev: TACGGTAGCAGAGACTTGGTCTAAATAACTTTTACRCCCCCACAAATA			

Underlined sequences indicate consensus sequences (CS1/CS2) for the addition of sequencing adapters

#### Singleplex bisulfite sequencing

PCR was carried out for each DNA sample as nine separate amplification reactions, each containing a single set of GR bisulfite sequencing primers. Primary reactions contained 5–10 ng bisulfite-converted DNA, 0.5 units HotStarTaq Plus DNA polymerase (Qiagen), 1X PCR buffer (Qiagen), 0.2 mM dNTPs, and 0.2 μM forward and reverse primers in a 25 μL volume. PCR conditions were 95 °C for 10 min followed by 40 cycles of denaturing at 95 °C for 30 s, annealing at 55 °C for 45 s, elongation at 72 °C for 30 s, followed by a final extension at 72 °C for 7 min. Secondary PCR for sample barcoding was performed in 50 μL reactions using 2 μL primary PCR product, 0.5 U Qiagen HotStarTaq Plus (Qiagen), 1X PCR buffer (Qiagen), 0.2 mM dNTPs, 3 mM MgCl2, and 0.08 μM of forward (barcoded) and reverse sequencing primers. PCR conditions were 95 °C for 15 min followed by 5 cycles of denaturing at 95 °C for 30 s, annealing at 58 °C for 30 s, elongation at 72 °C for 30 s, followed by a final extension step at 72 °C for 7 min. Equal volumes of secondary PCR products from each GR bisulfite sequencing primer set were pooled together by sample and purified with Agencourt AmpureXP magnetic beads (Beckman Coulter). Sample libraries were combined in equimolar concentrations and then sequenced on an Ion Torrent platform.

#### Multiplex bisulfite sequencing

PCR was carried out for each DNA sample as a single reaction containing the nine GR bisulfite primer sets. Primary reactions contained 10 ng bisulfite converted DNA, 1X Multiplex PCR *Plus* Master Mix (Qiagen), and 0.8 μM multiplex primer mix (each primer), composed of GR bisulfite sequencing forward and reverse primers, in a 25-μL reaction volume. PCR conditions were 95 °C for 15 min followed by 35 cycles of denaturing at 95 °C for 30 s, annealing at 60 °C for 90 s, and elongation at 72 °C for 90 s, followed by a final extension at 68 °C for 10 min. Products were purified with Agencourt AmpureXP magnetic beads (Beckman Coulter) and eluted in 20 μL of water. Secondary PCR for sample barcoding was performed in 50 μL reactions as described above for singleplex library preparation, using 10 μL purified primary multiplex PCR product as a template. Samples were quantified by Qubit dsDNA High Sensitivity fluorometric assay (Invitrogen), pooled at equal concentrations, and then purified with Agencourt AmpureXP magnetic beads (Beckman Coulter) followed by Ion Torrent sequencing.

### Bisulfite sequencing data processing

Sequencing-generated BAM files for each sample were uploaded to the Galaxy web platform [28] for processing prior to methylation analysis. The sequence files were filtered to retain reads > 100 bp, converted to FASTA format, and separated by GR bisulfite sequencing primer set (demultiplexing). The resulting FASTA files were loaded into BiQ Analyzer HiMod [29], which was used to align the sequence reads to GR promoter reference sequences and determine the mean methylation level (on a scale from 0, completely unmethylated, to 1, completely methylated) of each GR promoter region. The filter settings for alignment and methylation calling were minimum sequence identity = 0.9, minimum bisulfite conversion rate = 0.95, and maximum fraction of unrecognized CpG sites = 0.1. Mean methylation values from GR promoter regions that did not pass the minimum sequencing quality threshold (minimum coverage 50×) were considered as missing values. In the MA.12 cohort, the T region failed to pass this threshold in 135 (64.9%) cases and was therefore excluded from further analysis. There were 27 patients with at least one missing value due to minimum coverage requirements; however, most of these patients were missing data for only 1 region and all but one patient had 3 or less.

### Statistical analysis

Publicly available level 3 RNAseq and Illumina HumanMethylation450K (HM50K) data from The Cancer Genome Atlas (TCGA) (https://cancergenome.nih.gov/) was obtained for 363 breast tumor samples with positive ER status and 98 normal breast samples from the breast invasive carcinoma [BRCA] dataset. Normalized relative GR mRNA expression levels were compared between groups using Student’s *t* test. Differences in methylation between sample groups were assessed by Mann-Whitney *U* test. The relationship between relative gene expression values and mean methylation values for HM450K probes throughout the GR promoter (Additional file 2: Table S2) was assessed by Spearman rank correlation. All statistical analyses with TCGA data were conducted using GraphPad Prism 6.0.

To assess the relationship between methylation values from the singleplex and multiplex assays, two-tailed Pearson correlation coefficients were calculated with GraphPad Prism 6.0. All other analyses were performed using SPSS version 25.0 (IBM Corp, 2017). OS and DFS were described by Kaplan-Meier curves, with the difference between groups compared by log-rank test. Cox proportional hazards models were also used to assess the impact of prognostic factors on OS and DFS, as well as multivariate Cox models adjusting for age, tumor stage, nodal status, type of chemotherapy, and treatment. Multivariate Cox models with the same covariates, including an additional interaction term between treatment and GR methylation, were used to assess predictive value in OS and DFS. The chi-squared test, or Fisher’s exact test if at least one category was equal to 5 or less, was used to evaluate the relationship between GR methylation and clinical characteristics. All statistical tests were two-sided and *P* values of < 0.05 were considered significant.

For analysis of GR expression levels in breast cancer microarray studies, Kaplan-Meier plotter [30] was used to generate Kaplan-Meier curves for all ER+ patients regardless of treatment and for ER+ patients treated with tamoxifen. The overall survival and relapse-free survival of patients with high versus low GR expression were compared. The JetSet best probe set for the GR gene *NR3C1*, 216321_s_at, and an optimized threshold for the cutoff between high and low GR expression was used for all analyses. Hazard ratio (with 95% confidence interval) and log-rank *P* values were calculated by Kaplan-Meier plotter.

## Results

### ER+ breast tumors exhibit reduced GR expression levels and increased promoter methylation compared to normal breast tissue

**Fig. 1:**
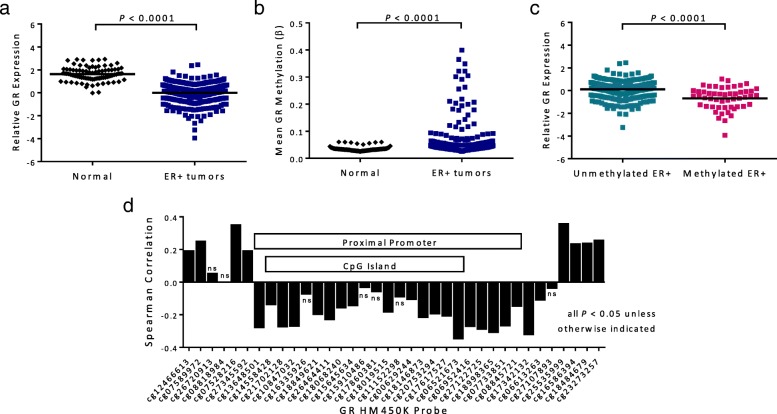
GR (*NR3C1*) expression and methylation in ER+ breast tumors and normal tissue from the TCGA breast cohort. **a** Relative GR mRNA expression levels in normal breast tissue and ER+ breast tumors from normalized RNAseq data. Black lines indicate mean GR expression. *P* value refers to Student’s *t* test. **b** Mean methylation of GR in normal tissue and ER+ tumor samples. Mean methylation calculated from beta-values for the 17 CpGs in the Illumina HumanMethylation450K array located within the GR proximal promoter CpG island. *P* value refers to Mann-Whitney *U* test. **c** Relative GR mRNA expression levels in ER+ tumors with (methylated ER+) and without (unmethylated ER+) GR CpG island methylation. ER+ tumors were divided into unmethylated versus methylated according to the highest mean GR CpG island methylation beta-value from normal breast tissue samples (*β* = 0.06). Black lines indicate mean expression. *P* value refers to Student’s *t* test. **d** Correlation of GR methylation and expression in ER+ tumors, at all 36 CpG sites across the GR gene included in the HM450K array. Methylation for all sites in the GR CpG island and proximal promoter region is correlated with reduced expression of GR mRNA levels, with Spearman correlation *P* < 0.05 unless otherwise indicated as not significant (ns)

The GR gene, *NR3C1*, has nine alternative first exons that are controlled independently by separate promoters [31, 32]. The first exon promoters that regulate GR expression in the breast are found within a 3-kb CpG island that spans the proximal promoter region of the gene and is susceptible to DNA methylation [24]. In a previous study, we showed that breast tumors had decreased GR expression compared to matched normal tissue and found that a subset of predominantly ER+ tumors exhibited methylation throughout the GR CpG island promoter region [24]. To further examine the role of GR methylation in ER+ breast cancer, we profiled GR expression and methylation in samples from the TCGA breast cohort. RNAseq and Illumina HumanMethylation450K (HM450K) data was obtained for 98 normal breast samples and 363 ER+ breast tumors. GR mRNA expression levels were significantly lower in ER+ tumors in comparison to normal breast tissue (*P* < 0.0001, Fig. 1a), consistent with previous findings. Methylation of the GR promoter was assessed for each sample based on the mean methylation (*β* values) of the HM450K probes (17 CpGs) within the GR CpG island. This analysis revealed significantly higher levels of GR promoter methylation in the ER+ tumors than in the normal breast tissue samples (*P* < 0.0001), which all exhibited low levels of methylation in this region (Fig. 1b). There were 52 ER+ tumors (14.3%) with GR methylation levels exceeding all normal samples, and this subset of GR-methylated ER+ tumors exhibited significantly reduced GR expression compared to the rest of the ER+ tumors (*P* < 0.0001, Fig. 1c). Next, the relationship between DNA methylation and gene expression in ER+ tumors was assessed at each HM450K probe within the GR gene. Results of the Spearman correlation showed that GR expression was significantly inversely correlated with methylation (*P* < 0.05) at 19 of 23 proximal promoter CpG sites, and the remainder of probes in the promoter displayed a negative trend (Fig. 1d). These results indicate that the expression of GR is frequently downregulated in ER+ by the aberrant methylation of its proximal promoter CpG island and suggests that many sites within the promoter region could be important for mediating this effect.

### Design of a targeted, bisulfite sequencing-based assay to detect GR promoter methylation

**Fig. 2:**
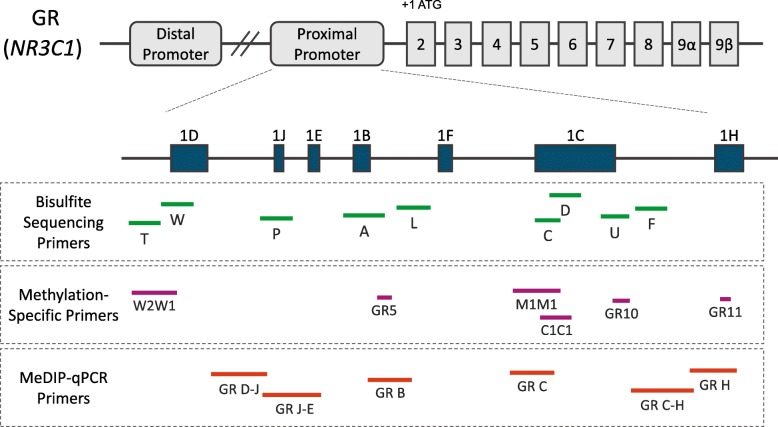
GR gene (*NR3C1*) proximal promoter with primer locations for bisulfite sequencing, MSP, and MeDIP-qPCR. The gene consists of eight coding exons (2-9α/β) and nine untranslated alternative first exons which are located in the distal and proximal promoter regions. The distal promoter is located approximately 30 kb upstream of the coding exons, and unlike the proximal promoter, it has minimal activity in the breast. The GR proximal promoter spans a 3 kb region approximately 5 kb upstream from the translation start site in exon 2 and contains seven alternative first exons (blue boxes). Primers for bisulfite sequencing (green bars depict the regions amplified by each primer set) were designed spanning the proximal promoter region and were used for our singleplex and multiplex GR bisulfite sequencing assay. MSP primer set regions are shown in purple and the location of qPCR primer sets for MeDIP-qPCR, performed in our previous study [24], are shown in orange. Genomic coordinates (hg19) for these regions are reported in Additional file 2: Table S2

To analyze GR promoter methylation in the MA.12 cohort, we designed a bisulfite sequencing-based assay that would be appropriate for working with the limited amounts and poor quality of material from archival formalin-fixed paraffin-embedded (FFPE) tumor. FFPE samples often yield only a limited amount of DNA that can be extensively fragmented [33]. Moreover, DNA is prone to degradation during the bisulfite conversion process, leading to further fragmentation of the sample and a reduction in the amount of amplifiable template for PCR [34]. To mitigate this, primer sets were designed to target short stretches of DNA (< 200 bp), which are less likely to contain strand breaks and more likely to be amplified successfully. Due to the high GC content of the GR proximal promoter and the reduced sequence complexity following bisulfite conversion, designing primers that were sufficiently specific was a challenge. By using degenerate primers (i.e., both C and T are present at the C of the CpG) containing up to 3 CpGs that are capable of annealing to the target region regardless of methylation status, we expanded the promoter regions that were suitable for primer placement. The result was an assay consisting of nine bisulfite PCR primer sets (T, W, P, A, L, C, D, U, and F) that each interrogates the methylation status of 7 to 17 CpGs in the GR proximal promoter (Fig. 2). Multiplexed PCR with the nine primer sets is performed to amplify the bisulfite-converted sample DNA, which is then followed by the next-generation sequencing (NGS) to evaluate GR methylation within the targeted promoter regions.

### Establishment of the GR bisulfite sequencing assay as a functional test of GR methylation in breast cell line controls

**Fig. 3:**
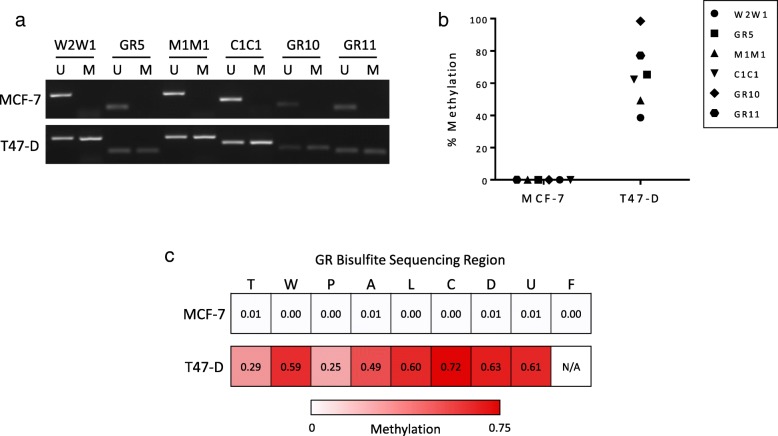
GR promoter methylation analysis in MCF-7 and T47-D breast cell lines. **a** Endpoint MSP products for unmethylated (U) and methylated (M) MSP primer sets in MCF-7 and T47-D cells. **b** qMSP analysis of GR promoter methylation. Samples that generated product with only the unmethylated-specific primer set were considered completely unmethylated. **c** Heat map of mean methylation levels for each GR promoter region, detected by singleplex bisulfite sequencing

The GR bisulfite sequencing assay was initially tested using two ER+ breast cell lines, MCF-7 and T47-D, which had their GR methylation status determined by MeDIP-qPCR in our earlier study [24]. To further validate these previous findings, we performed quantitative methylation-specific PCR (qMSP). In agreement with the MeDIP-qPCR results, MCF-7 cells did not show any GR promoter methylation, whereas T47-D cells were methylated at each of the assayed promoter regions (Fig. 3a,b). Therefore, MCF-7 and T47-D cells were used as the negative and positive controls for GR methylation, respectively.

The performance of the nine GR bisulfite sequencing primer sets was first evaluated individually (singleplex) in the two cell line controls, prior to multiplexing. MCF-7 cells were unmethylated throughout the GR promoter (methylation ≤ 0.01 for all regions), and T47-D cells were methylated, with each primer set reporting a methylation value of at least 0.25 (Fig. 3c). This was consistent with the qMSP data, indicating that the individual primer sets were detecting GR methylation effectively. Although the F primer set region did not meet the minimum sequencing coverage requirements in the T47-D sample, because it performed well in MCF-7 cells, it was still included for testing in the multiplex version of the assay.

**Fig. 4:**
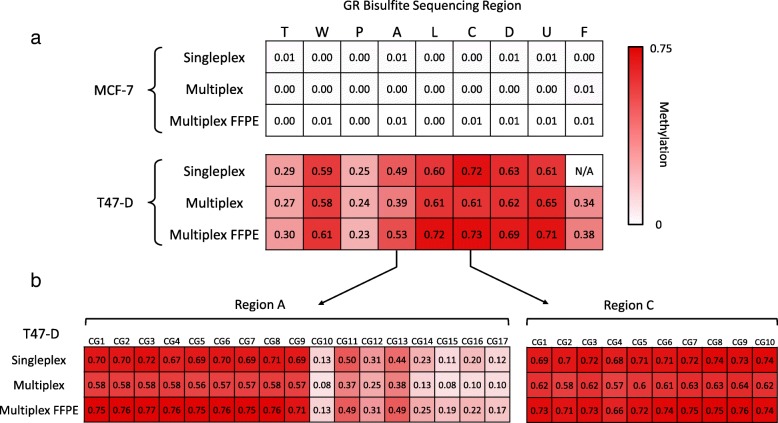
Singleplex and multiplex GR bisulfite sequencing in MCF-7 and T47-D cell lines and FFPE cell pellets. GR bisulfite sequencing was performed as a singleplex assay (separate reactions for each primer set) or a multiplex assay (all primer sets used in a single reaction) for cell lines and as a multiplex assay with DNA extracted from formalin-fixed paraffin-embedded cell pellets (Multiplex FFPE). **a** Heat map of mean methylation levels for each bisulfite sequencing region in the GR promoter. **b** Heat map of mean methylation level for each CpG contained within GR bisulfite sequencing region A (17 CpGs) and region C (10 CpGs) in T47-D cells

Next, the GR bisulfite sequencing assay was tested with MCF-7 and T47-D cells using the nine primer sets in a multiplex reaction. DNA extracted from FFPE MCF-7 and T47-D cell pellets was also tested to investigate assay performance in FFPE samples. As anticipated, the pattern of GR methylation from the singleplex bisulfite sequencing was similarly reproduced in both the cell line and cell pellet FFPE samples tested by the multiplex assay (Fig. 4a). This was also seen at the level of individual CpG sites contained between each primer pair. For example, in many cases, all CpG sites in an assayed region were either methylated or unmethylated, as was seen for the C primer set region; however, there were several examples in T47-D cells, such as the A region, where the pattern of methylation was more varied (Fig. 4b). These methylation patterns were conserved in the multiplex results from cell lines and in the FFPE cell pellets. This suggested that despite the fragmentation that occurs with the fixation and embedding process, the GR bisulfite sequencing assay was robust and would be applicable to DNA from FFPE samples.

### Multiplex GR bisulfite sequencing assay limit of detection

**Fig. 5:**
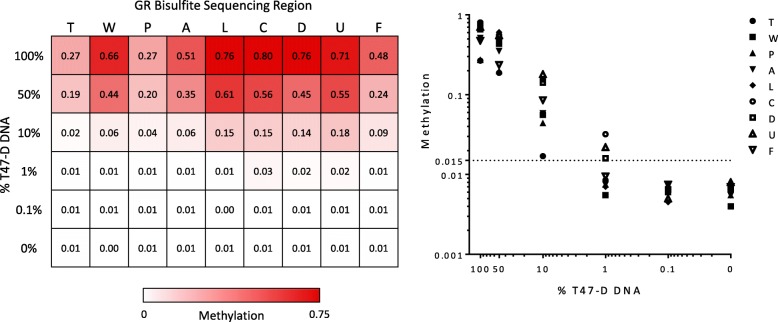
Multiplex GR bisulfite sequencing assay detection limit. T47-D (GR-methylated) DNA was added in varying amounts (10 to 0.01 ng) to a fixed amount (10 ng) of MCF-7 (GR-unmethylated) DNA. These mixtures (50%, 10%, 1%, and 0.1% T47-D DNA) and samples containing T47-D only (100%) and no T47-D DNA (0%, MCF-7 DNA only) were used to test the ability of the multiplex bisulfite sequencing assay to detect GR-methylated DNA in a background of unmethylated DNA. DNA from MCF-7 cells was assayed multiple times and was used to set a threshold for methylation which was defined as three standard deviations above the mean methylation level of all GR bisulfite sequencing regions (0.015, indicated by a dotted line). The primer sets for regions C, D, and U could detect as little as 1% T47-D DNA with this cutoff, and all could detect the sample with 10%

Tumor samples typically contain a mixture of cancerous and normal cells. Therefore, a dilution experiment was done to determine the ability of the multiplex bisulfite sequencing assay to detect GR-methylated tumor DNA against a background of DNA from GR-unmethylated cells. Varying amounts of T47-D DNA (10 to 0.01 ng) were added to a fixed 10 ng of DNA from MCF-7 cells to represent samples with a range of methylated tumor cells. A sample containing only MCF-7 DNA was assayed multiple times and used to set a methylation threshold for the primer sets in the assay (methylation ≥ 0.015), defined as three standard deviations above the mean methylation of all regions. With this cutoff, all primer sets in the multiplex assay detected 1 ng of methylated T47-D DNA in a background of 10 ng unmethylated DNA, and the C, D, and U primer sets could detect as little as 0.1 ng (Fig. 5). Thus, samples with from 10% to as little as 1% methylated tumor cells would still be classified as methylated by the GR bisulfite sequencing assay.

### Validation of GR bisulfite sequencing assay in breast tumor samples

**Fig. 6:**
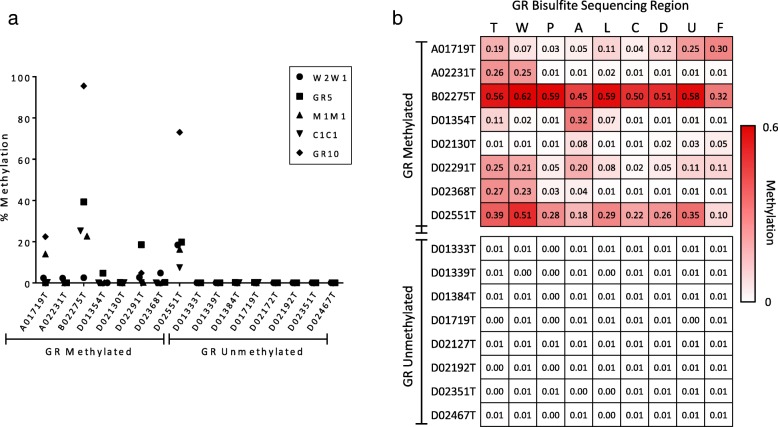
GR promoter methylation analysis in breast tumor samples with known GR promoter methylation status. **a** qMSP analysis of GR promoter methylation. Samples that generated product with only the unmethylated-specific primer set were considered completely unmethylated. **b** Heat map of mean methylation levels for each GR promoter region, detected by multiplex bisulfite sequencing

Tumors are composed of a heterogeneous population of cells, including stromal and immune cells, and as a result, they are more complex than cell lines. Therefore, prior to testing the MA.12 cohort, the GR bisulfite sequencing assay was validated with DNA extracted from breast tumor samples. A small cohort of fresh frozen breast tumors from the Ontario Tumour Bank had been tested with MeDIP-qPCR as part of our previous study to determine their GR methylation status [24]. Here, we examined a subset of these tumors that included both GR-methylated and GR-unmethylated samples. As with the MCF-7 and T47-D cell line controls, the GR methylation status of the tumors was confirmed by qMSP (Fig. 6a).

To ensure that the results from the singleplex and multiplex bisulfite sequencing assay remained consistent in this more complex sample type, several of the breast tumors were tested with both assay preparation methods and a Pearson correlation was used to assess the association between the results from each method. As anticipated, there was a strong positive correlation between the methylation values generated by both tests, for all the primer set regions. The correlations were statistically significant with all greater or equal to *r* = 0.97, *P* < 0.0001, with *R*^*2*^ = 0.942 (Additional file 6: Figure S1).

Next, the multiplex assay was used to assess GR methylation in eight GR-methylated and eight GR-unmethylated tumors (Fig. 6b). Overall, the results were in accordance with those from qMSP. GR-unmethylated tumor samples, similar to MCF-7 cells, lacked methylation at all of the interrogated primer regions (methylation ≤ 0.015 for all regions). In contrast, the GR-methylated tumors all had two or more promoter regions with methylation ≥ 0.05. In comparison to the methylation pattern observed in T47-D cells, methylation in the breast tumors was more spatially varied across the GR promoter, with several samples only exhibiting methylation at a subset of the interrogated promoter regions (Fig. 6b). However, this was not unexpected as it was reflective of the previous MeDIP-qPCR findings and was also apparent in the qMSP results. A single sample, D02130T, despite previously being identified as methylated by MeDIP-qPCR was not classified as methylated by the qMSP assay, yet results from singleplex and multiplex bisulfite sequencing both detected low levels of methylation at primer regions A and F. Further investigation at the CpG level revealed that methylation in this sample was localized to several small areas that did not coincide with the limited number of CpGs assessed by our qMSP probes. Altogether, analysis of the fresh frozen breast tumor samples further supports the integrity of the GR bisulfite sequencing assay and demonstrates its performance in a more complex sample type.

### GR methylation as a prognostic marker in ER+ breast cancer patients from the MA.12 clinical trial cohort

The CCTG MA.12 study recruited 672 breast cancer patients, with 338 randomized to tamoxifen and 334 to placebo. There were 454 MA.12 patients with ER+ breast cancer, and 252 ER+ FFPE tumor samples were available for DNA extraction, 208 of which yielded enough material for bisulfite conversion and subsequent methylation assessment by multiplex bisulfite sequencing. The characteristics of all ER+ patients from the MA.12 trial (*n* = 454) and the patients in the GR methylation study cohort (*n* = 208) are described in Table 1. The clinicopathological characteristics were similar between the two cohorts. There were 56 deaths (26.9%) and 86 relapse events (41.3%) that occurred within the 208-patient GR methylation study cohort during the 9.7-year median follow-up time of the MA.12 trial. To investigate the clinical significance of GR promoter methylation in these patients, eight GR promoter regions were assessed by multiplex bisulfite sequencing (the T promoter region was excluded from analysis due to low sequencing coverage), methylation at each region was dichotomized according to the previously determined MCF-7-derived threshold, and the results were compared with patient overall survival (OS) and disease-free survival (DFS).

**Fig. 7:**
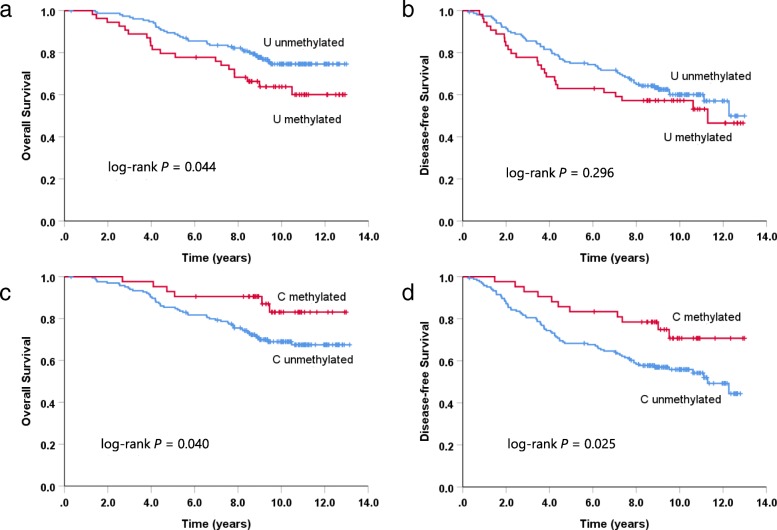
Kaplan-Meier curves of overall survival (**a**, **c**) and disease-free survival (**b**, **d**) according to methylation of GR bisulfite sequencing region U (**a**, **b**) and methylation of GR bisulfite sequencing region C (**c**, **d**) in the MA.12 cohort. *P* values were derived from log-rank tests

Univariate survival analyses performed for each of the eight GR promoter regions indicated that region C and region U were both potential prognostic factors for patient outcome in the whole methylation study cohort (Table 3, Fig. 7). Patients with methylation of region U had significantly worse OS (HR = 1.742, 95% CI 1.008–3.011; *P* = 0.047, Fig. 7a) compared to those without methylation in the U region. U region-methylated patients also had a poorer 5-year DFS rate (63.0% of patients relapse-free at 5 years versus 75.0% for unmethylated patients), although the methylation status of this region did not significantly impact DFS overall (HR = 1.282, 95% CI 0.803–2.046; *P* = 0.289, Fig. 7b). Methylation of the C region was also associated with significant differences in patient outcome; however, unexpectedly, methylation in this region was associated with better OS (HR = 0.423, 95% CI 0.181–0.986; *P* = 0.046, Fig. 7c) and DFS (HR = 0.492, 95% CI 0.261–0.927; *P* = 0.028, Fig. 7d). Of the 86 relapse patients in the study cohort, 25 (29%) had tumors with U region methylation and 11 (13%) had C region methylation, including one patient with both U and C methylation (Additional file 3: Table S3). In the subset of patients who died (*n* = 56), U region methylation accounted for an even greater proportion of patients (20 patients, 36%) while instances of C region methylation remained relatively low (6 patients, 11%). Multivariate analysis, carried out for U and C region methylation separately, confirmed that these regions remained prognostic indicators of outcome independent of patient age, pathological stage, T-stage, nodal status, adjuvant chemotherapy, and treatment (placebo versus tamoxifen) (Table 3). Methylation of the remaining GR promoter regions was not associated with statistically significant differences in either OS or DFS (Table 3).

**Table 3:** Cox proportional hazards analysis of disease-free survival and overall survival for MA.12 GR methylation study patients

Characteristic	OS		DFS	
	Hazard ratio [95% CI]	*P*	Hazard ratio [95% CI]	*P*
Univariate analysis				
GR methylation				
A	0.869 [0.445–1.698]	0.682	0.807 [0.464–1.403]	0.448
C	0.423 [0.181–0.986]	*0.046*	0.492 [0.261–0.927]	*0.028*
D	0.867 [0.437–1.722]	0.684	1.155 [0.692–1.928]	0.580
F	0.816 [0.457–1.457]	0.491	0.707 [0.438–1.140]	0.154
L	1.403 [0.750–2.625]	0.289	1.470 [0.886–2.438]	0.136
P	1.059 [0.518–2.164]	0.875	1.249 [0.725–2.155]	0.423
U	1.742 [1.008–3.011]	*0.047*	1.282 [0.803–2.046]	0.289
W	1.294 [0.716–2.339]	0.393	1.227 [0.755–1.997]	0.409
Age		0.089		0.178
≤ 45 years	1		1	
> 45 years	1.577 [0.933–2.666]		1.340 [0.875–20.53]	
Stage (pathological)		0.8		0.534
I	1		1	
II	1.536 [0.380–6.428]	0.536	1.724 [0.543–5.470]	0.355
III	1.352 [0.247–7.381]	0.728	2.136 [0.566–8.055]	0.262
Pathological T-stage		0.094		0.261
T1	1		1	
T2	1.891 [1.059–3.377]	*0.031*	1.418 [0.903–2.228]	0.130
T3/T4	1.310 [0.440–3.898]	0.627	1.552 [0.713–3.379]	0.268
Nodal status		*0.008*		*0.007*
Node negative	1		1	
1–3 nodes	1.071 [0.446–2.571]	0.878	1.679 [0.763–3.696]	0.198
4–9 nodes	1.357 [0.515–3.572]	0.537	1.832 [0.769–4.362]	0.172
10+ nodes	4.863 [1.565–15.109]	*0.006*	6.285 [2.106–18.754]	*0.001*
Adjuvant chemotherapy		0.31		0.366
CEF	1		1	
CMF	0.840 [0.446–1.583]	0.59	0.794 [0.473–1.332]	0.382
AC	0.570 [0.274–1.186]	0.133	0.662 [0.373–1.174]	0.158
Treatment		0.914		0.489
Placebo	1		1	
Tamoxifen	1.029 [0.609–1.741]		0.860 [0.561–1.318]	
Multivariate analysis				
GR methylation				
C	0.396 [0.164–0.957]	*0.04*	0.492 [0.252–0.957]	*0.037*
U	1.786 [1.025–3.113]	*0.041*	1.318 [0.822–2.115]	0.252

Multivariate Cox proportional hazards models are adjusted for age, pathological stage, pathological T-stage, nodal status, type of adjuvant chemotherapy, and treatment arm. *P* values < 0.05 are shown in italics

To further characterize the clinical significance of GR methylation in breast cancer, we examined the relationships between the methylation status of each promoter region and clinicopathological characteristics which are already known survival predictors including age, pathological stage, T-stage, and nodal status (Additional file 4: Table S4). Methylation of the C region and lower nodal involvement almost reached significance (*P* = 0.064), while L region methylation was associated with both higher pathological stage (*P* = 0.013) and higher nodal status (*P* = 0.023). Univariate survival analysis of clinicopathological characteristics demonstrated that the nodal status was a strong predictor of OS and DFS in our cohort and that higher T-stage (T2 vs T1) was associated with worse OS (Table 3).

**Fig. 8:**
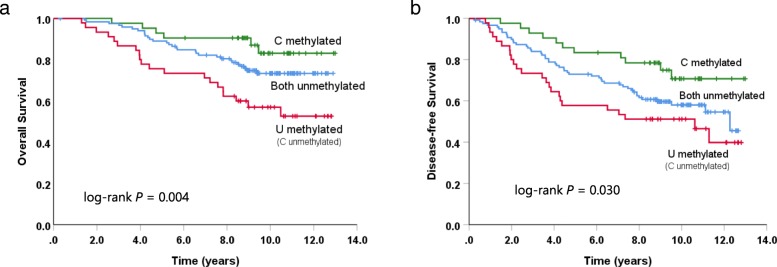
Methylation of the GR bisulfite sequencing C and U regions and patient outcome. Kaplan-Meier curves of overall survival (**a**) and disease-free survival (**b**) according to the C and U region methylation groups. Patients methylated at both C and U are included in the C methylated group (green). *P* values were derived from log-rank tests

The identification of two regions of the same promoter with opposite associations with OS and DFS is unusual. Given our expectation that GR methylation would in general be associated with worse outcome, one of the simplest explanations is that the region C methylation is actually related to a lack of methylation over the rest of the promoter. Logistic regression analysis of the methylation status of these regions indicated that methylation of region C was inversely correlated with methylation in regions W, A, L, and U (*P* < 0.05) supporting the idea that the better outcomes are due to decreased methylation across the rest of the promoter. There were 42 patients with methylation of region C and 54 with methylation of region U, but only 9 patients with both C and U region methylation indicating the two regions were mostly mutually exclusive. Of the 9 patients positive for both C and U, 5 were not methylated in regions W, A, or L suggesting the patients with C and U methylation were more similar to region C methylated tumors. Therefore, patients were divided into three groups according to the methylation status of the C and U regions: C methylated (*n* = 42), U methylated without C methylation (*n* = 45), and no methylation in either region (*n* = 119). Univariate Cox proportional hazards models showed that both OS (*P* = 0.006) and DFS (*P* = 0.035) were statistically significantly different across these GR methylation groups (Table 4), with the C region methylation group having the best outcomes and the U methylation without C methylation group having the worst (Fig. 8a, b). There was no significant association between these GR methylation groups and patient clinicopathological characteristics (Additional file 5: Table S5).

**Table 4:** Cox proportional hazards analysis of disease-free survival and overall survival for GR methylation groups based on C and U region methylation status

Characteristic	OS		DFS	
	Hazard ratio [95% CI]	*P*	Hazard ratio [95% CI]	*P*
Univariate analysis				
GR methylation group		*0.006*		*0.035*
Both C and U unmethylated	1		1	
C methylated	0.524 [0.218–1.260]	0.149	0.549 [0.286–1.054]	0.072
U methylated, no C methylation	2.008 [1.140–3.537]	*0.016*	1.407 [0.864–2.291]	0.170
Multivariate analysis				
Age		0.083		0.152
≤ 45 years	1		1	
> 45 years	1.624 [0.939–2.810]		1.389 [0.886–2.178]	
Stage (pathological)		0.985		0.976
I	1		1	
II	1.054 [0.167–6.672]	0.955	0.901 [0.179–4.529]	0.899
III	0.731 [0.005–107.941]	0.902	0.653 [0.015–27.996]	0.824
Pathological T-stage		0.542		0.558
T1	1		1	
T2	1.448 [0.751–2.790]	0.269	1.313 [0.788–2.189]	0.296
T3/T4	1.401 [0.015–129.717]	0.884	1.830 [0.073–46.030]	0.713
Nodal status		*0.022*		*0.029*
Node negative	1		1	
1–3 nodes	1.052 [0.334–3.308]	0.932	1.638 [0.540–4.972]	0.383
4–9 nodes	1.153 [0.337–3.945]	0.82	1.569 [0.488–5.048]	0.45
10+ nodes	5.007 [1.201–20.875]	*0.027*	6.004 [1.494–24.129]	*0.012*
Adjuvant chemotherapy		0.517		0.624
CEF	1		1	
CMF	1.018 [0.493–2.103]	0.961	0.944 [0.519–1.718]	0.851
AC	0.698 [0.319–1.526]	0.367	0.755 [0.399–1.426]	0.386
Treatment		0.552		0.136
Placebo	1		1	
Tamoxifen	0.844 [0.484–1.474]		0.710 [0.452–1.115]	
GR methylation group		*0.006*		*0.041*
Both C and U unmethylated	1		1	
C methylated	0.504 [0.203–1.255]	0.141	0.559 [0.280–1.115]	0.099
U methylated, no C methylation	1.988 [1.112–3.553]	*0.02*	1.431 [0.866–2.365]	0.162

Multivariate Cox proportional hazards models are adjusted for age, pathological stage, pathological T-stage, nodal status, type of adjuvant chemotherapy, and treatment arm, as shown. *P* values < 0.05 are shown in italics

In multivariate analysis adjusting for age, pathological stage, T-stage, nodal status, adjuvant chemotherapy, and treatment, GR methylation group remained an independent prognostic factor for OS and DFS (Table 4), with the same relationship between groups as observed in the univariate analysis. Patients with U methylation, but no C methylation had markedly worse OS compared to patients with no C or U region methylation (HR = 1.988, 95% CI 1.112–3.553; *P* = 0.02) and compared to patients in the C methylated group (HR = 3.941, 95% CI 1.540–10.085; *P* = 0.004). U methylation group patients also had an increased risk of relapsing compared to patients with C methylation (HR = 2.560, 95% CI 1.226–5.343; *P* = 0.012). Although it did not reach statistical significance, there was a trend towards better OS (HR = 0.504, 95% CI 0.203–1.255; *P* = 0.141) and DFS (HR = 0.559, 95% CI 0.280–1.115; *P* = 0.099) in the patients with C methylation compared to those with unmethylated C and U regions. Altogether these results suggest that methylation of the C and U bisulfite sequencing assay regions within the GR CpG island promoter have significant prognostic value in this cohort of ER+ breast cancer patients.

Since the MA.12 trial was used to validate PAM50 [35], we used the data available from that analysis to examine if our GR methylation groups based on C and U region methylation were related to any of the intrinsic PAM50 subtypes. Luminal B breast cancers for example have a poor prognosis compared to luminal A tumors and also have been associated with a particular methylation signature (CpG Island Methylator Phenotype, CIMP) [36, 37]. PAM50 data was available for 165 tumors in our study cohort. As expected, most were luminal A (46.7%) and luminal B (28.5%), with some HER-2 enriched (18.2%) and several basal (3.0%) and normal (3.6%) samples. There was no statistically significant difference in the distribution of PAM50 subtypes between our three GR methylation groups (*P* = 0.327). Similarly, the risk of recurrence (ROR) scores, which are generated from PAM50 subtype data alone (RORS) or together with proliferation signature index (RORP) or tumor size (RORT), was also not significantly different between GR methylation groups (Table 5), suggesting that GR methylation is not associated with the PAM50 signature.

**Table 5:** Comparison of GR methylation groups with PAM50 characteristics

Characteristic	C methylated		Both C and U unmethylated		U methylated, no C methylation		χ^2^	*P*
	*N*	%	*N*	%	*N*	%		
PAM50 subtype								
Luminal A	16	44.4	48	51.6	13	36.1	8.602	0.327
Luminal B	14	38.9	22	23.7	11	30.6		
HER2	5	13.9	15	16.1	10	27.8		
Basal	1	2.8	4	4.3	0	0.0		
Normal	0	0.0	4	4.3	2	5.6		
RORS group								
Low	12	33.3	38	40.9	14	38.9	2.667	0.615
Medium	18	50.0	41	44.1	13	36.1		
High	6	16.7	14	15.1	9	25.0		
RORP group								
Low	10	27.8	26	28.0	6	16.7	3.475	0.482
Medium	17	47.2	52	55.9	21	58.3		
High	9	25.0	15	16.1	9	25.0		
RORT group								
Low	8	22.2	26	28.0	7	19.4	1.581	0.812
Medium	19	52.8	48	51.6	19	52.8		
High	9	25.0	19	20.4	10	27.8		

PAM50 data was available for 165 samples

### GR methylation as a predictive marker of tamoxifen benefit in ER+ breast cancer patients from the MA.12 clinical trial cohort

**Fig. 9:**
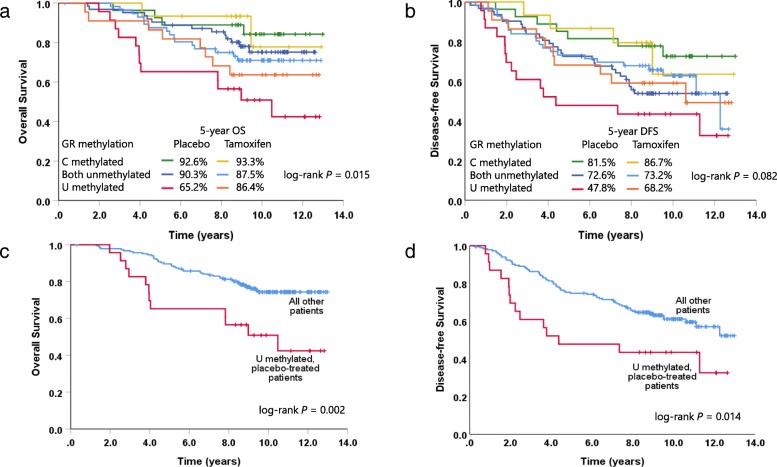
Effect of tamoxifen treatment on patients according to GR methylation group. Kaplan-Meier curves show overall survival (**a**) and disease-free survival (**b**) with patients grouped by treatment arm (placebo versus tamoxifen) and by methylation status of GR bisulfite sequencing regions C and U. Survival at 5 years from treatment arm randomization is shown for each group. Kapan-Meier curves comparing overall survival (**c**) and disease-free survival (**d**) between placebo-treated patients in the U methylated, C unmethylated group (red) compared to all other patients in the study cohort (blue). *P* values were derived from log-rank tests

Even though the complete MA.12 trial was marginally positive for the effect of tamoxifen on DFS (HR = 0.77, *P* = 0.06) and not significant for its effect on OS (HR = 0.78, *P* = 0.12), we analyzed the relationship between the C and U region methylation groups and the effect of tamoxifen treatment (Fig. 9a, b, Table 6). Within the patients with U but not C methylation, there appeared to be a greater trend for benefit from tamoxifen, both in terms of OS (adjusted HR = 0.414, 95% CI 0.142–1.203; *P* = 0.105) and DFS (adjusted HR = 0.430, 95% CI 0.166–1.110; *P* = 0.081), although this did not reach significance. No significant benefit for tamoxifen was found in the other GR methylation groups (C methylation, and no C or U methylation) and the interaction between GR methylation group and tamoxifen treatment was not significant for OS (*P =* 0.427) or DFS (*P* = 0.723). Despite this, dividing patients according to both GR methylation group and treatment arm may have prognostic utility. While the U methylated, C unmethylated group of patients had worse outcomes overall, those also in the placebo arm had particularly poor OS (adjusted HR = 2.855, 95% CI 1.462–5.575; *P* = 0.002, Fig. 9c) and DFS (adjusted HR = 2.091, 95% CI 1.159–3.771; *P* = 0.014, Fig. 9d), when compared to the rest of the cohort as a whole. However, the small numbers of patients involved means these results should be treated with caution.

**Table 6:** Predictive analysis of GR methylation groups

GR methylation group	Treatment	# of patients	OS				DFS			
			5-year OS	Hazard ratio [95% CI]	*P*	*P value for interaction*	5-year DFS	Hazard ratio [95% CI]	*P*	*P value for interaction*
C methylated	Placebo	27	92.6%	1	0.82	0.427	81.5%	1	0.789	0.723
	Tamoxifen	15	93.3%	0.744 [0.058–9.598]			86.7%	1.221 [0.266–5.597]		
Both C and U unmethylated	Placebo	62	90.3%	1	0.596		72.6%	1	0.328	
	Tamoxifen	57	87.5%	1.258 [0.563–2.493]			73.2%	0.755 [0.418–1.361]		
U methylated, no C methylation	Placebo	23	65.2%	1	0.105		47.8%	1	0.081	
	Tamoxifen	22	86.4%	0.414 [0.142–1.203]			68.2%	0.430 [0.166–1.110]		

Hazard ratios are adjusted for age, pathological stage, pathological T-stage, nodal status, and type of adjuvant chemotherapy

### Decreased GR expression in ER+ breast cancer correlates with poor clinical outcome in an independent cohort

**Fig. 10:**
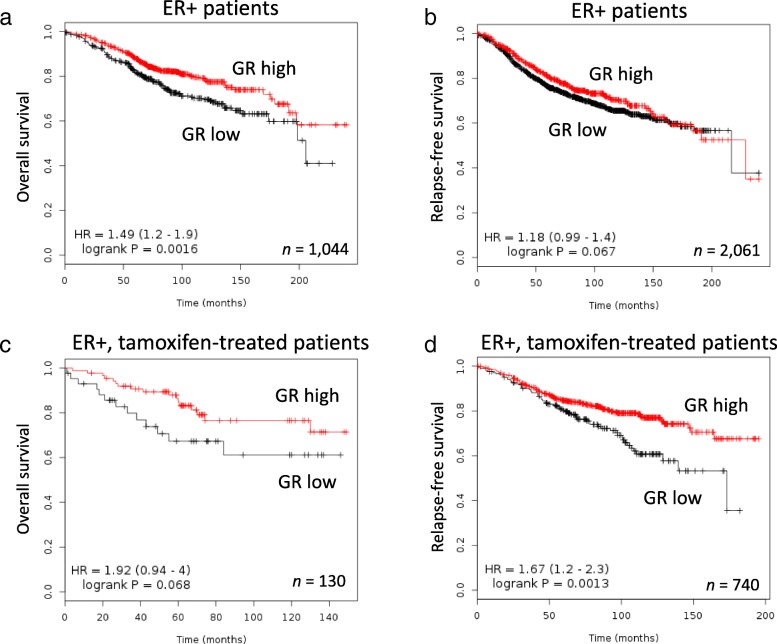
Kaplan-Meier curves of overall survival (**a**, **c**) and disease-free survival in ER+ breast cancer patients from the KM plotter cohort divided by GR mRNA expression level. ER+ patients regardless of treatment received (**a**, **b**) or treated with tamoxifen (**c**, **d**) were grouped by high or low GR expression using an optimized cutoff. The JetSet probe for GR (*NR3C1*, 216321_s_at) was used for all analyses. Hazard ratios and log-rank *P* values were calculated by KM plotter. The number of patients (*n*) in each analysis is indicated

We expanded our investigation of GR as a marker of poor outcome in an independent cohort using the online survival analysis tool, Kaplan-Meier plotter [30]. The multi-study breast cancer cohort assembled by Kaplan-Meier plotter contains gene expression data with extended patient survival information. The relationship between GR mRNA expression level (according to an optimized cutoff) and outcome was assessed for all ER+ patients, regardless of treatment regimen (Fig. 10a, b). Patients with low GR-expressing tumors exhibited an increased risk of breast cancer-related death (*P* = 0.0016) compared to those with high GR expression levels. A similar trend was observed with respect to the risk of relapse; however, it did not quite reach statistical significance (*P* = 0.067). Decreased GR expression levels also appeared to be detrimental in ER+ patients treated with tamoxifen (Fig. 10c, d), as it was marginally associated with poor OS (*P* = 0.068) and significantly associated with increased relapse risk (*P* = 0.0013). Given that decreased GR expression is an expected consequence of GR promoter methylation, these findings further support a role for GR methylation as a marker of poor outcome in ER+ breast cancer patients.

## Discussion

As part of this study, we have developed a multiplexed next-generation sequencing protocol for the analysis of DNA methylation that optimizes the small quantities of nucleic acid material available from FFPE tissue for molecular-level analysis while limiting the cost of this type of analysis. This technique was effective for the assessment of DNA from fresh frozen tumor samples as well as material from the MA.12 clinical trial where the FFPE samples were from 18 to 25 years old. The ability to interrogate completed clinical trials is a singular advantage for characterizing the predictive power of specific biomarkers both within the context of the specific agents being tested but also in general. Access to archival samples in the form of FFPE tumor material allows for the interrogation of protein, DNA, and RNA from these trials which often have many years of follow-up, which is in contrast to new comprehensive analyses such as TCGA in which follow-up is extremely limited [38]. Indeed, MA.12 was used to validate the PAM50 assay [35], which has come into clinical practice as the Prosigna test [39]. The value of DNA methylation biomarkers is increasingly recognized [40] and has distinct technical advantages in the context of FFPE material. Methylation markers are often highly tumor-specific and effect multiple CpGs, and as such do not suffer as extremely from the cellularity issues that limit both RNA quantification and DNA mutation analyses. Here, for example, we have demonstrated that levels of tumor DNA as low as 1% can be easily distinguished from normal DNA. Our approach also provides access to methylation analysis at the nucleotide level, interrogating more CpGs (7 to 17) than qMSP (generally 2–3). This has advantages when methylation is spatially complex, as seen with sample D02130T, which was negative by qMSP but had localized methylation that was revealed by sequencing. For this study, we have analyzed 9 different sites across the highly complex glucocorticoid receptor proximal promoter, but this technique could just as easily be applied to 9 or more different regions across the genome.

DNA methylation markers in breast cancer are recognized as having the potential to aid in the management of patients by providing significant prognostic information [41]. Our observation of both positive and negative associations between methylation at the same gene is unusual in that most markers tend to be one or the other [41]. The relatively mutually exclusive nature of C region and methylation of other regions and particularly the U region may explain this inverse association. Furthermore, methylation at these two regions appears to have a differential effect on GR expression, as preliminary immunohistochemical staining of MA.12 tissue microarrays has revealed that GR staining (H-scores) in U methylated tumors is around 3-fold lower than tumors with C methylation (data not shown). Previous studies of GR expression and function in breast cancer have revealed a similarly complex situation involving both increased and decreased risk. In Triple-Negative Breast Cancer (TNBC), higher levels of GR expression have been associated with poor outcome [18]. This is thought to stem from the protective effect of activation of the stress response wherein the upregulation of SGK-1 by GR may lead to resistance to apoptosis [42]. More recently, GR activation has been observed in breast cancer metastases and was associated with a GR activation signature which included genes such as MELK, CDK1, and particularly the ROR1 kinase, which may mediate resistance to chemotherapeutic agents and increase colonization [43]. This phenotype was associated particularly with the claudin-low group [43], which is a subtype of TNBC [44]. Our ER classification was based on the original MA.12 biochemical or immunohistochemistry assessment but was largely confirmed by the PAM50 intrinsic subtype analysis and included only 5 patients with a basal phenotype. Of these, only 1 displayed C region methylation, and none had U region methylation; thus, our markers are unlikely to be associated with a TNBC phenotype.

In contrast with TNBC, in ER+ breast cancer, higher levels of GR mRNA are associated with good outcome [13, 18]. This may be associated with the induction of differentiation mediated through increased co-occupancy by ER and GR at multiple sites in the genome [13]. Similarly, the potential for GR to participate in the suppression of ER+ breast cancer proliferative gene expression by occupying ER-bound enhancers has also been recently described [45]. We have previously defined a tumor suppressor-like activity of the unliganded GR which activates multiple targets that may also result in the induction of differentiation, apoptosis, and growth arrest [46]. The transcriptional activation of BRCA1 expression in particular may be a target for this unliganded GR function [47]. Loss of GR expression through promoter methylation, and thus, of these tumor-suppressive GR functions, could contribute to breast cancer development and progression by allowing for increased cell survival and potentially increased tumor growth from a loss of GR-mediated regulation of ER signaling. We have also suggested that GR methylation could be linked to psychological stress [24] providing a link between stress and the etiology of breast cancer [48].

U region methylation was found in over 25% of patients, while C region methylation was found in 20% of patients, and with the two being mostly mutually exclusive, they provide an assessment in almost half of our patient cohort. MA.12 is also a relatively young cohort with a median age of 46 years (range 29–58) representing a pre- and peri-menopausal population where tamoxifen would be expected to be more effective [3]. Neither U or C methylation was associated with the PAM50 assessment of intrinsic phenotype indicating that their prognostic ability is not the result of an association of C with the more favorable luminal A subtype or U with the less favorable luminal B subtype. GR promoter methylation could be assessed in many samples where the original PAM50 test failed (43 out of 208), though it has now been updated to use NanoString technology [39]. The commercial test, Prosigna, is typically is used to stratify patients into risk groups where those with the lowest risk are spared aggressive treatment [49]. The C region biomarker could serve as an ancillary marker and being based on DNA methylation, which we have demonstrated to be very robust and resistant to normal contamination, may be applicable to samples that are otherwise unable to be assessed, for example, due to RNA degradation. In contrast, patients with U methylation who did not receive tamoxifen had particularly early relapse and death which was only partially improved with tamoxifen treatment. This may reflect a need for GR function for effective anti-estrogen action [13] or may identify tumors that have already gained estrogen independence [50]. This would be an intrinsic, rather than acquired property of the tumor, as it was assessed in the primary tissue. These patients could be treated more aggressively in an attempt to compensate for the ineffectiveness of anti-estrogen therapy.

This MA.12 cohort was comprised of relatively young breast cancer patients (60% less than 45 years old) but the TCGA and KM plotter data all point to these results being applicable to older women as well. The confirmation of this work will require validation in additional cohorts that are more reflective of the overall pre- and post-menopausal breast cancer populations.

## Conclusions

Our results suggest that in ER+ breast cancers, the status of the GR gene, as reflected by promoter methylation, may define a previously uncharacterized subset of patients and that GR methylation status plays an important role in determining the response to treatment and ultimately determines the prognosis of some of these patients.

## Supplementary information

**Additional file 1: Table S1.** Primers for quantitative Methylation-Specific PCR (qMSP).

**Additional file 2: Table S2.** Genomic coordinates for all GR promoter methylation primers and probes.

**Additional file 3: Table S3.** GR methylation in U and C regions for patients by outcome.

**Additional file 4: Table S4.** Association of GR methylation and clinical characteristics in MA.12 GR methylation study patients.

**Additional file 5: Table S5.** Association of GR methylation groups (based on methylation of C and U promoter regions) and clinical characteristics in MA.12 GR methylation study patients.

**Additional file 6: Figure S1.** Comparison of GR methylation values generated by singleplex and multiplex GR bisulfite sequencing assay methods in breast tumor samples. DNA from fresh frozen breast tumors (*n* = 11) was tested by both the singleplex and the multiplex GR bisulfite sequencing assay and the methylation values for each primer set in the assay were compared using a Pearson correlation. There was a strong positive correlation between the two tests and all correlations were statistically significant with greater or equal to *r* = 0.97, *P* < 0.0001. *R*^*2*^ values for the relationship between methylation values are shown for each primer set.

## Data Availability

The datasets analyzed here are available from the TCGA Research Network (available at: http://cancergenome.nih.gov/) and Kaplan-Meier plotter (available at: http://kmplot.com/analysis/). All other data generated and/or analyzed during the current study are available from the corresponding author on reasonable request.
