# Supplementary figures and images for: Region-specific glucocorticoid receptor promoter methylation has both positive and negative prognostic value in patients with estrogen receptor-positive breast cancer
Source: Clin Epigenetics. 2019 Nov 1;11:155. doi: 10.1186/s13148-019-0750-x (PMC6825343; doi:10.1186/s13148-019-0750-x)

## Slide 1
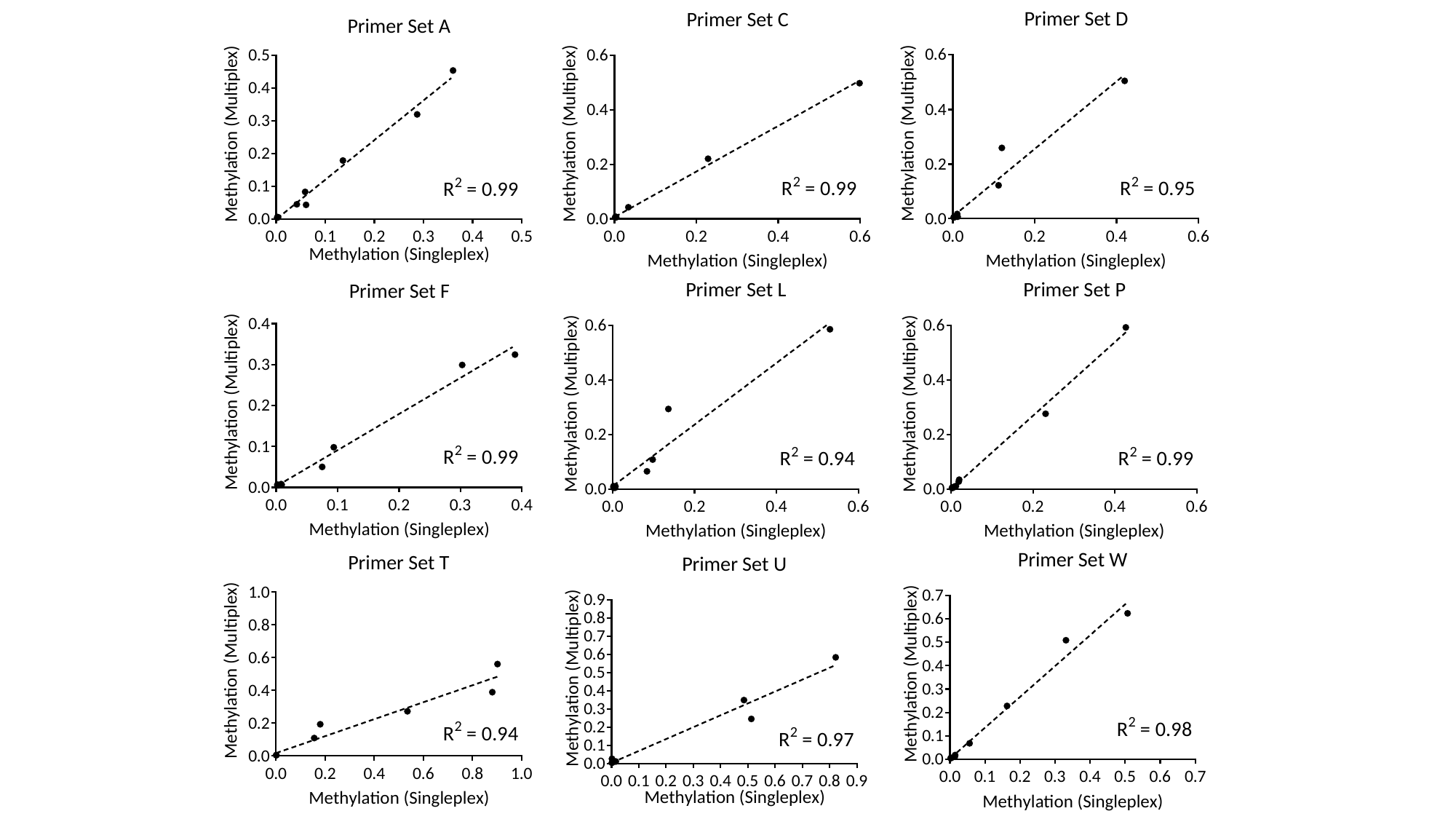

Supplement: Supplementary file 6 — Additional file 6: Figure S1. Comparison of GR methylation values generated by singleplex and multiplex GR bisulfite sequencing assay methods in breast tumor samples. DNA from fresh frozen breast tumors (n = 11) was tested by both the singleplex and the multiplex GR bisulfite sequencing assay and the methylation values for each primer set in the assay were compared using a Pearson correlation. There was a strong positive correlation between the two tests and all correlations were statistically significant with greater or equal to r = 0.97, P < 0.0001. R2 values for the relationship between methylation values are shown for each primer set. [file 13148_2019_750_MOESM6_ESM.pptx]
